# Supporting Nursing Staff During Crises: Impact of Organisational Support Measures and Resources in Job Satisfaction in German Nursing Homes

**DOI:** 10.3390/geriatrics9060159

**Published:** 2024-12-11

**Authors:** Elisabeth Diehl, Anna Hirschmüller, Aline Wege, Albert Nienhaus, Pavel Dietz

**Affiliations:** 1 Institute of Occupational, Social and Environmental Medicine, University Medical Center of the Johannes Gutenberg University Mainz, Obere Zahlbacher Str. 67, 55131 Mainz, Germany; 2 Department for Occupational Medicine, Hazardous Substances and Health Science, Institution for Statutory Accident Insurance and Prevention in the Health and Welfare Services [BGW], Pappelallee 33/35/37, 22089 Hamburg, Germany

**Keywords:** nurses, job satisfaction, support measures, personal resources, social resources, COVID-19 pandemic

## Abstract

Background/Objectives: The COVID-19 pandemic placed an immense burden on nursing home staff, significantly increasing their workload. How the impact of these challenges on job satisfaction is mitigated by personal and social resources, along with organisational support measures initiated by nursing homes, is investigated in this study. Methods: In 2021, a cross-sectional survey was conducted among nursing home staff in Rhineland-Palatinate (n = 373). The questionnaire contained parts of standardised instruments (parts of the Copenhagen Psychosocial Questionnaire (COPSOQ), Brief Resilience Scale) and self-developed questions related to support measures such as training, psychological support and work organisation changes. The association of these support measures, as well as personal and social resources (e.g., resilience, social support, sense of community), with job satisfaction was explored. Descriptive, bivariate and regression analyses were conducted. Results: While various support measures were offered to the nursing home staff, significant gaps remained. Training on hygiene and COVID-19 care was beneficial but not universally available. Similarly, psychological, pastoral and palliative support was lacking for a large portion of nursing home staff. Surprisingly, in the regression analysis, frequent information updates from supervisors were found to be negatively correlated with job satisfaction (*p* = 0.002). However, some personal and social resources (resilience (*p* = 0.002), social support (*p* = 0.001), sense of community at work (*p* ≤ 0.001), commitment to the workplace (*p* = 0.019), recognition by management (*p* ≤ 0.001)), and various support measures (training programmes (*p* = 0.005), changes in work organisation (*p* = 0.008), technical measures (*p* = 0.025)) were positively correlated with job satisfaction. Conclusions: This study highlights that despite the implementation of various support measures for nursing home staff during the COVID-19 pandemic, significant gaps remained. Notably, a substantial portion of staff members lacked access to crucial support services such as psychological, pastoral and palliative care. Furthermore, healthcare facility managers should prioritise the following support measures, especially during crises: comprehensive training, including resilience training; flexible working arrangements; and recognition for nursing staff. Ultimately, healthcare organisations should aim to create a supportive work environment that fosters a sense of community and belonging among their nursing workforce.

## 1. Introduction

Nurses are important to society because they help to ensure the health and well-being of people. They not only help people when they need care, but also make an important contribution to the quality of life and well-being of society. Against the background of the current nursing shortage, greater importance should be given to the job satisfaction of nursing staff. There was a connection between nurses’ job satisfaction and their intention to leave the profession (nurse turnover intention) [1], even before the COVID-19 pandemic. Moreover, international studies have shown a correlation between high job satisfaction and lower turnover, better care quality, and higher patient satisfaction [2,3].

Nursing homes were particularly affected by COVID-19 due to the high number of elderly residents with pre-existing conditions and weakened immune systems [4]. This led to nurses in nursing homes experiencing not only an increased risk of infection but also having to cope with changes in work processes and working conditions, staff shortages [5] and individual stressors, such as the impacts of school and nursery closures on their child care responsibilities. Additionally, nursing home staff often experienced agonising circumstances, including frequent deaths of residents, which led to higher levels of emotional stress [6]. These challenges for nursing staff prompted the need for comprehensive support.

In response to the increasing demands and workloads faced by nursing staff, many institutions have taken a proactive approach by implementing targeted support measures [7]. Managers and supervisors occupied a special position during the COVID-19 pandemic, as they were responsible for communicating all relevant information to their employees in an understandable and up-to-date manner [8,9]. To minimise the risk of infection, personal protective equipment (PPE) was made available to nursing staff in nursing homes, although there were some initial delivery difficulties. This equipment included masks, gloves and protective gowns or face shields. Various active training procedures (including video or computer simulations or spoken instructions), and passive training strategies (lectures) were used to illustrate the correct handling of PPE [10]. Additionally, attempts were made to recruit additional staff or to reallocate existing staff in order to use them in other areas of work where there was a more urgent need for staff [11]. Governments announced an increase in care workers’ wages and offered financial incentives [12], for example, the so-called “Corona bonus” in Germany [13]. Moreover, psychological support services and training on coping with stress and emotional strain were also offered by governments [14] or health care institutions [15].

Nursing staff in nursing homes have various resources at their disposal, including work resources, such as support from colleagues, superiors or management [16,17]. These work resources are significantly associated with high job satisfaction [18,19]. Studies show that, particularly in the crisis context of the COVID-19 pandemic, social support in the workplace was considered a crucial, relevant resource for maintaining healthcare workers’ job satisfaction [20,21]. Resilience, as a personal resource, is defined as the process of successfully adapting to significant sources of stress or the ability to bounce back or recover from stress [22]. Study results suggest that resilience enables nurses to maintain their mental and psychological health, particularly in times of crisis [23]. Furthermore, there is evidence that interventions that increase healthcare workers’ resilience can reduce the risk of burnout, improve mental health [24] and job satisfaction [25], and reduce turnover intentions [26].

The COVID-19 pandemic has significantly increased the already high workload of nursing staff. In the context of high job demands, the presence of resources such as resilience or social support can contribute to the emergence of job satisfaction, while high job demands without sufficient resources can lead to job dissatisfaction [20]. While previous studies have tended to focus on single measures or resources, this study aims to provide a comprehensive overview of the support provided in nursing homes during the COVID-19 pandemic and to simultaneously examine a wide range of support measures. This study investigates, on the one hand, which organisational support measures were offered to nursing staff in nursing homes during the COVID-19 pandemic and which support measures were desired by the nursing staff themselves. On the other hand, the influence of these support measures, as well as the influence of personal and social resources on the job satisfaction of nursing staff, is also examined. The aim is to develop a better understanding of the specific needs of nursing staff in crisis situations in order to derive recommendations for action for future crises. In addition, this work has practical implications for employers on how to support nursing staff in nursing homes to improve their job satisfaction, not only in crisis situations.

## 2. Materials and Methods

### 2.1. Study Design

This cross-sectional study was conducted in 2021. Participation was voluntary and anonymous. Written informed consent was obtained from all participants at the beginning of the questionnaire. Approval to perform the study was received from the ethics committee of the State Chamber of Medicine in Rhineland-Palatinate (clearance number 2020-15537).

### 2.2. Study Procedure

We conducted a comprehensive internet search to identify all nursing homes with long-term care located in Rhineland-Palatinate. Of the 506 researched facilities, a 25% (126 facilities) random sample was initially constructed. The study team contacted all facilities in the sample by email and telephone.

Contacts started at the end of September 2021 and ended at the beginning of December 2021. The most frequently cited reasons for refusing by facility or nursing service management were that the healthcare staff did not have time and should not be additionally burdened.

### 2.3. Study Participants

To obtain accurate results for a finite population of approximately 26,000 nurses in nursing homes in Rhineland-Palatinate [27], with a 95% confidence level and a margin of error of 5%, we required a minimum sample size of 379 [28]. Assuming a response rate of 20%, at least 1895 questionnaires needed to be sent out in order to fulfil the required sample size.

Facility or nursing service managers who agreed to participate were asked for the number of nursing staff working in their facilities, and the corresponding number of paper questionnaires was sent to the facilities by post. The facility managers were then asked to distribute the questionnaires to the nursing staff. To facilitate easy return, a stamped, self-addressed envelope was included with each questionnaire. This allowed nursing staff to complete and return the questionnaire at their convenience. To protect participant anonymity, no personally identifiable information was collected.

The nursing staff were included in the study by the participating facilities. The study team had no direct contact with the nursing staff at any time.

### 2.4. Questionnaire

Based on the results of a qualitative pre-study [7,29], a questionnaire was designed. The questionnaire contained items related to sociodemographic data, professional information and various questions from validated instruments. The instruments used for the present study were the Copenhagen Psychosocial Questionnaire (COPSOQ), the Brief Resilience Scale (BRS) and self-developed questions for support measures.

The COPSOQ questionnaire included parts of the German standard version of the Copenhagen Psychosocial Questionnaire (COPSOQ), Version III. The COPSOQ is a valid and reliable questionnaire for the assessment of psychosocial work environmental factors and health in the workplace [30]. The following scales were utilised in this study:

- “Job satisfaction” (7 items, for example: “Regarding your work in general. How pleased are you with your work prospects?” with the answers being “very satisfied”, “satisfied”, “partly-satisfied”, “dissatisfied” or “very dissatisfied”)
- “social support” (4 items, for example: “How often do you get help and support from your colleagues?” with the answers being “always”, “often”, “sometimes”, “rarely”, “never/almost never”)
- “sense of community at work” (2 items, for example: “Is there a good atmosphere between you and your colleagues?” with the answers being “always”, “often”, “sometimes”, “rarely”, “never/almost never”)
- “commitment to the workplace” (2 items, for example: “Are you proud of being part of this organisation?” with the answers being “to a very high degree”, “to a high degree”, “to some extent”, “to a low degree”, “to a very low degree”)
- “recognition by management” (1 item: “Is your work recognised and appreciated by the management?” with the answers being “to a very high degree”, “to a high degree”, “to some extent”, “to a low degree”, “to a very low degree”)

The original version of the Brief Resilience Scale (BRS) was developed by Smith et al. [22] to assess resilience. The German version of the BRS is a short six-item measure in which the following is asked: “Please indicate the extent to which you agree with each of the following statements by using the following scale: 1 = strongly disagree, 2 = disagree, 3 = neutral, 4 = agree, 5 = strongly agree”. An example item is: “I tend to bounce back quickly after hard times”. The German adaption of the BRS is a reliable and valid instrument for measuring resilience [31].

Questions based on the results of the qualitative pre-study [7] about organisational support measures were developed. These questions were subsequently pretested and finally incorporated into the questionnaire. The following section presents the self-developed questions and their respective response categories:

#### 2.4.1. Support from People (Outside the Facility)

Question: During the pandemic, did the following (external) people take on tasks in your facility? If so, which groups of people were they, and how helpful did you find them? The following answer categories were available: (1) Students/apprentices, (2) agency workers/temporary workers, (3) Bundeswehr personnel, (4) volunteers and (5) former colleagues with the answer categories “yes—offered and helpful”, “yes—offered but not helpful”, “not offered but would have desired it” and “not offered and I didn’t need it”.

#### 2.4.2. Support Measures

Question: Please indicate which of the following measures were offered in your facility during the pandemic and, if they were not, whether you would have desired these measures. The following support measures were evaluated:

- Internal staff support: Two questions regarding the provision of personnel support through personnel reallocation and recruitment of new personnel.
- Training programmes: Four questions on advanced training/education on the implementation of hygiene measures, education/training on how to use protective equipment, advanced training/education on handling COVID-19 patients and education/training on dealing with relatives.
- Changes in work organisation: Two questions related to changes in the work organisation and home office, if applicable (e.g., for documentation work).
- Psychological support: Two questions regarding supervision and psychological support.
- Pastoral and palliative support: Two questions on pastoral support in dealing with death and dying and palliative care/medical support.

Additionally, single questions were added to gather information about COVID-19 measures implemented by superiors, in-house financial support, relaxation of hygiene measures for vaccination protection, possibilities for communication between residents and relatives, and technical measures (e.g., use of air filters). The following answer categories were used to evaluate these questions: “yes—offered and helpful”, “yes—offered but not helpful”, “yes—offered but I did not use it”, “not offered but I would have desired it”, and “not offered and I didn’t need it”.

### 2.5. Data Preparation

Scales selected from the COPSOQ were prepared according to COPSOQ guidelines. Generally, COPSOQ items have a five-point Likert format, with scores then converted to a 0–100 scale. This transformation is a standardised procedure and conforms to the German COPSOQ validation study. The scale score is calculated as the mean of the items for each scale if at least half of the single items had valid answers. Data on nursing home staff members who answered less than half of the items on a scale were recorded as “missing”. If at least half of the items were answered, the scale value was calculated as the average score of the items answered [32]. High scale values of the scales used in the present paper were considered positive. Cronbach’s alpha was used to evaluate the internal consistency of the scales, with a value >0.7 considered acceptable [33]. The negatively phrased items of the BRS were reversed in order to calculate the mean of the six BRS items [31].

In Section 3, descriptive results are presented regarding the basic characteristics of the study population, the COPSOQ scales and the single organisational support measures. The number of measures collected in each support category is also reported as part of the descriptive analysis.

For further analysis, the support categories as well as the single questions were dichotomised [either a measure was offered or not].

### 2.6. Data Analysis

The dependent variable in our study (job satisfaction) is continuous. We used Pearson correlations to examine the relationship between two continuous variables. For comparisons involving one continuous and one binary variable, we conducted t-tests. When one variable was continuous and the other categorical with more than two levels, we utilised analysis of variance (ANOVA). All variables found to be significantly related to job satisfaction in bivariate analyses were further examined in a regression analysis. As past research has shown that resilience and social resources are significant predictors of job satisfaction, we decided to perform a hierarchical regression, in which variables were entered in blocks. In the first step, demographic and job-specific variables were entered as confounder variables. Participants who stated that they had a qualification as a geriatric nurse or a nurse or a university degree in the field of nursing were grouped into one category for the regression analysis (i.e., the qualified degree category). In step 2, resilience (as a metric variable) was added to the model. In step 3, work-specific social resources (COPSOQ scales as metric variables) were entered into the model. In the fourth and final step, further organisational support measures were added to the model (as binary variables “not offered” vs. “offered”). The assumptions for the regression analyses (linearity, independent errors, homoscedasticity, multicollinearity and normally distributed residuals) were checked [33]. Using a hierarchical regression model, we examined the unique contributions of nurses’ resources and support measures to job satisfaction after controlling for demographic and job-specific variables. The significance level was set at *p* < 0.05 (two-tailed). Statistical analyses and graphical representations were performed using SPSS version 29.0 and Microsoft Excel 2016, respectively.

## 3. Results

Of the 2448 questionnaires sent to 55 institutions, 404 were returned (response rate = 16.5%). After data cleaning, the final number of participants was 373.

### 3.1. Descriptive Results

Study population

The basic characteristics of the study population are presented in Table 1. The mean age of the nursing staff was 42.6 years, and the majority were female. About half of those surveyed had a qualification as a nurse, 18% had a qualification as a nursing assistant, and one-third were in training, had another type of educational qualification or reported no education. About a quarter had a leadership position.

Support from people (outside the institution)

**Figure 1:**
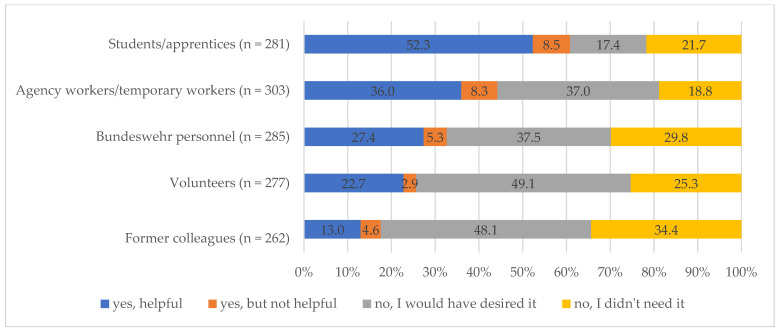
Support from people (outside the institution).

A substantial majority of nursing staff (61%) received assistance from students or apprentices, and more than half (52%) found this support valuable in strengthening the team’s capacity. Among those who did not receive support from students or apprentices, a notable proportion (17%) expressed a desire for such assistance. Similarly, agency or temporary workers provided aid to a considerable number of nursing staff (44%), with approximately one-third finding this support beneficial. However, a similar proportion of nursing staff (37%) indicated a preference for receiving support from agency or temporary workers. Bundeswehr personnel deployed by the government to assist healthcare facilities were reported as helpful by a portion of the nursing staff (27%), while an even larger proportion (38%) wished for their assistance. Notably, around half of the respondents expressed a desire for additional support from volunteers and former colleagues. Regarding volunteers, 23% of nursing staff found their assistance helpful, while 13% reported benefiting from the support of former colleagues. A substantial proportion of nursing staff (between 19% and 35%) indicated no need for external support (Figure 1).

Organisational support measures during the COVID-19 pandemic in the facilities

**Figure 2:**
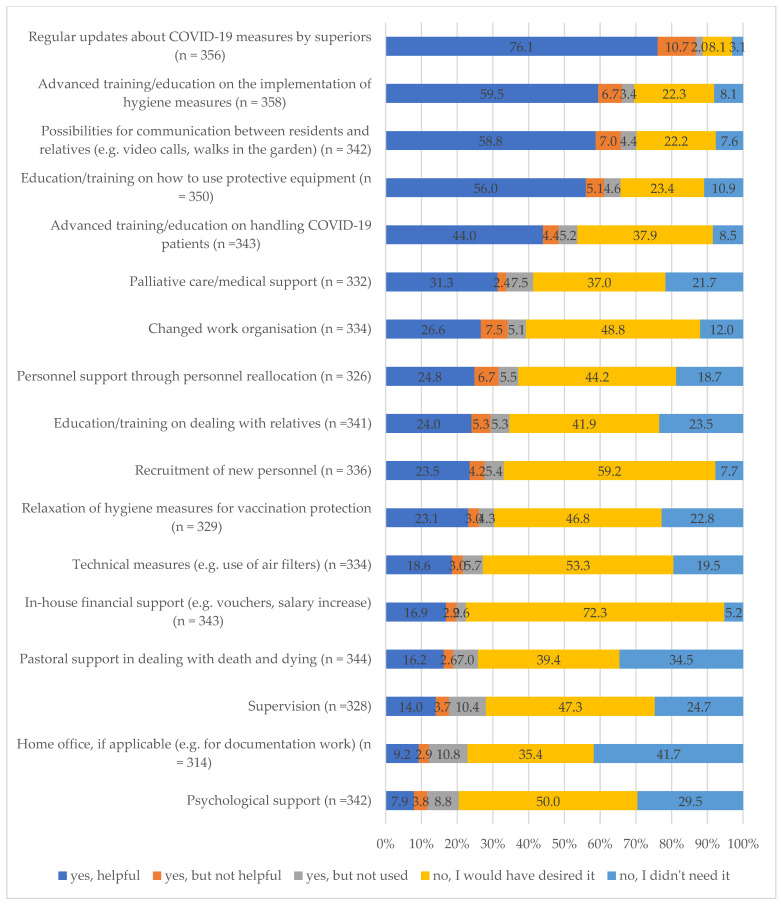
Support measures.

The majority of respondents appreciated the regular communication from their supervisors regarding COVID-19 measures (Figure 2). While 76% found this information valuable, 11% considered it unhelpful, and 8% desired more frequent updates. Regarding advanced training on hygiene measures, nearly 70% of nursing staff received such training, with the majority finding it beneficial. However, 30% of nursing staff lacked access to such training, expressing a desire for it. Similarly, over half of the respondents received advanced training on handling COVID-19 patients, finding it helpful. However, 46% of nursing staff reported no such training, with the majority wishing for it. Concerning training on dealing with relatives of COVID-19 patients, one-third of nursing staff received this type of training, finding it beneficial. However, 42% of nursing staff expressed a desire for such training.

Nearly 60% of nursing staff lacked access to support offers for palliative care or medical assistance. Similarly, over 70% of nursing staff reported no support regarding pastoral guidance in dealing with death and dying. Furthermore, a significant proportion of nursing staff (72%) indicated no access to supervision, and an even larger proportion (80%) received no psychological support. Notably, when these support options were available, most nursing staff found them beneficial (Figure 2).

About three-quarters (70%) of the nursing staff reported having possibilities for communication between residents and relatives, and most of them found this helpful. About 60% wished for the recruitment of new personnel. In facilities where this was done, most of the nursing staff (24%) reported it as helpful. Similar results were found regarding the change in work organisation and personnel support through personnel reallocation. More than 70% of the nursing staff reported the desire for some in-house financial support.

Upon examining the organisational support measure categories (as outlined in Section 2.4.1 and Section 2.4.2), it is evident that more than half of the nursing staff reported not having received psychological or pastoral and palliative support. In addition, more than half of the nursing staff reported that no changes were made to the work organisation or that there was no internal staff support, such as a redistribution of staff or the hiring of new staff. About one-quarter of the respondents reported that their facility did not offer personnel support from external persons or different training programmes. Similarly, about a quarter of the respondents reported receiving training in all areas (Supplementary Figure S1). Nursing staff reported receiving an average of 8 of the 22 support measures they were asked about. The range of responses was 0 to 22, with a standard deviation of 4.8.

Table 2 presents the mean scores and standard deviations for the COPSOQ scales “job satisfaction”, “social support”, “sense of community at work”, “commitment to the workplace” and “recognition by management”. Additionally, the number of scale items and the internal consistency (Cronbach’s alpha) are listed. All COPSOQ scales in this study showed satisfactory internal consistency. Mean resilience was 3.1 (SD 0.6), with a range of 1.33–5.00. The reliability of the scale was moderate (Cronbach’s alpha = 0.646).

### 3.2. Bivariate Analyses

There was no association between the basic and job-related characteristics of the sample presented in Table 1 and job satisfaction. Further, no association was found between job satisfaction and support provided by external persons. With regard to organisational support measures within the organisation, a significant association with job satisfaction was evident for all support measures surveyed (Table 3). The correlations between job satisfaction and the COPSOQ scales used, as well as resilience, are presented in Table 4.

### 3.3. Results of Regression Analysis

The hierarchical regression was conducted with four blocks of variables. The first block included age, sex, professional qualification and leadership position as the predictors, with job satisfaction as the dependent variable. In block two, resilience was included, and in block three, further job resources (social support, sense of community at work, commitment to the workplace and recognition by management) were included. In block four, organisational support offers were also included, with job satisfaction as the dependent variable.

Overall, the results showed that the first model was not significant (Table 5), while the second model, which included resilience, was significant (F(8, 207) = 5.356, *p* < 0.001, R^2^ = 0.171). The third model, which included social resources, showed a significant improvement from the second model (F(12, 203) = 23.671, *p* < 0.001, R^2^ = 0.583, change R^2^ = 0.412). The fourth model showed further significant improvement when adding support measures to the model (F(22, 193) = 15.828, *p* < 0.001, R^2^ = 0.643, change R^2^ = 0.060). Overall, when resilience was included in the model, the variables explained 13.9% of the variance. The inclusion of further job resources in the model explained 55.9% of the variance, and the final model, including organisational support offers, accounted for 60.3% of the variance. The results show that age, gender, professional qualification and leadership position were not significant predictors of job satisfaction, while resilience, social support, sense of community at work, commitment to the workplace, recognition by management, the offer of training programmes, changes in working organisation and the implementation of technical measures were significant predictors of job satisfaction. For example, if training programmes were offered, job satisfaction increased by an average of 5.9 points on a scale from 0 to 100. The measure of regular updates about COVID-19 measures implemented by superiors was a negative predictor of job satisfaction.

## 4. Discussion

Nursing staff have a demanding job that requires considerable physical and emotional effort. Nursing home staff often care for older, demented and frail patients with complex psychosocial and medical needs. Many studies have shown that the workload of nursing home staff increased both physically and psychologically during the COVID-19 pandemic [5,6,7]. This made it even more important for nursing homes to provide support measures for their staff.

This paper shows the status quo of support measures in nursing homes in Rhineland-Palatinate at a time when the COVID-19 pandemic had been ongoing for about two years. The results illustrate that various measures were taken to relieve the burden on nursing staff in nursing homes during the pandemic. These measures included the use of additional staff (internal, external, volunteers, Bundeswehr personnel), training programmes, psychological and palliative or pastoral support, and changes to the work organisation. Overall, support was offered, but the descriptive results show that more could have been done. For example, regarding advanced training on hygiene measures, nearly 70% of nursing staff received such training, with the majority finding it beneficial. However, nearly one-third of nursing staff lacked access to such training, expressing a desire for it. Similarly, more than half of the respondents received advanced training on handling COVID-19 patients, finding it helpful. However, nearly half of the nursing staff reported no such training, with the majority wishing for it. Concerning training on how to deal with relatives of COVID-19 patients, one-third of nursing staff received such training, finding it beneficial, while more than 40% of nursing staff did not and expressed a desire for such training. Surprisingly, a quarter of nursing staff reported not having received any offer for training on the topics asked about in the survey.

The psychological and emotional strain placed on nursing home staff was brought to the forefront during the pandemic, exacerbated by the high number of COVID-19 deaths. The study findings reveal that 60 to 80% of the respondents reported receiving no support in dealing with death, whether in psychosocial, palliative or post-mortem care. This revelation is particularly striking given the advancements and growing recognition of palliative care in recent years [34]. In palliative care, frequent and painful deaths are part of nurses’ daily work. Nevertheless, palliative care workers appear to be less stressed than nursing staff in other areas. They are also more satisfied with their work [17]. Not only in the wake of future crises, but also in general, employers of nursing staff in nursing homes should rely on the support potential of palliative care to provide relief for their employees.

The findings of the current study align with the results of previous research, which have consistently demonstrated a positive association between training programmes and enhanced job satisfaction [35,36]. In light of these findings, employers should consistently engage in proactive efforts to understand and address the needs of their employees, irrespective of crisis situations. This proactive approach should include providing appropriate training and development opportunities to foster a more engaged and satisfied workforce.

The authors generally believe that regular communication from employers can help to reduce employee uncertainty and anxiety, ultimately boosting motivation and team spirit. Intriguingly, the multivariate analysis revealed that frequent updates on COVID-19 measures disseminated by supervisors negatively correlated with job satisfaction. This finding could be interpreted within the framework of the Conservation of Resources (COR) theory [37]. While regular communication can generally serve as a resource by reducing uncertainty, an overload of information, especially during crises, can lead to the depletion of psychological resources. The constant need to adapt to new guidelines and the associated workload may decrease employees’ perceived control, acting as additional stressors. This is in line with the results of the pilot study, where nursing staff reported feeling overwhelmed by the constant stream of government regulations and the associated workload [7]. This suggests that, paradoxically, receiving regular updates from supervisors may actually contribute to lower job satisfaction among nursing staff. Further research is needed to explore this paradoxical finding and identify the optimal frequency and content of communication from supervisors to promote employee well-being and job satisfaction in nursing homes, regardless of crisis situations. For example, this includes a more differentiated consideration of the quality of communication and the individual needs of employees in order to develop effective communication strategies for everyday work. Future studies should therefore focus more strongly on the quality of communication. Aspects such as the content, relevance, clarity and type of information provided (e.g., written, oral, interactive) could have a decisive influence on job satisfaction [38]. Future research efforts are crucial for gaining a deeper understanding of the nuances of healthcare communication and developing effective strategies. Qualitative studies can examine the perspectives of nursing staff on how they experience and perceive communication from their supervisors. Quantitative studies can evaluate the effects of different communication strategies on employee satisfaction and other relevant outcomes. By understanding the nuances of communication in healthcare settings, we can develop effective strategies to enhance employee engagement and resilience during times of crisis.

The results of the present study also show that nursing staff who report having personal and social resources experience significantly higher levels of job satisfaction. This study specifically examined the following personal and social resources: resilience, social support, sense of community at work and commitment to the workplace. This finding aligns with previous research, which has consistently emphasised the profound influence of personal and social resources on job satisfaction among healthcare workers [39,40,41]. Previous studies have also reported a positive relationship between resilience and job satisfaction [25,26]. Furthermore, the results of this study are consistent with those of a recent study, which found that recognition from superiors is a significant predictor of job satisfaction [42]. While the current study focused specifically on job satisfaction, a wealth of other research has demonstrated that various resources can also positively impact physical and mental well-being, enhancing the overall health and well-being of healthcare professionals [42,43,44,45,46]. These findings underscore the crucial role of personal and social resources in promoting the job satisfaction of nursing staff, particularly in high-stress environments like nursing homes.

Ultimately, the results of this study confirm findings from the severe acute respiratory syndrome (SARS) outbreak in 2003. For example, a comprehensive analysis of 22 studies during the SARS outbreak underscored the effectiveness of supportive work environments, adequate training and overall organisational preparedness in protecting healthcare workers against the detrimental mental health consequences of such crises [47].

### Limitations

The survey instrument used in the present study included valid and reliable instruments, such as parts of the COPSOQ and the BRS. Additionally, it included self-developed questions, which were not validated but were valuable for our study, as they addressed certain topics that standardised questionnaires could not. In order to achieve transparency, we reported the frequency of each self-developed question. The geographical scope of this study was limited to Rhineland-Palatinate, precluding generalisations to the whole of Germany or other countries. Additionally, the response rate of 16.5% is considered relatively low. The most common reason for non-participation of nursing homes was the high workload of the nursing staff. It is conceivable that nursing homes that participated in the study have more resources or a stronger interest in the research topic. This could lead to a positive bias in the results, as these institutions may have already implemented more successful measures to improve working conditions, which can subsequently affect job satisfaction. To enhance response rates in future studies, researchers should consider establishing direct contact with nurses. Furthermore, longitudinal studies should be conducted to observe changes over time, identify causal relationships, and analyse individual developmental trajectories. Due to the cross-sectional design of the survey, it is not possible to analyse changes in job satisfaction over time or to establish causal relationships.

## 5. Conclusions

The study corroborates existing findings and offers additional insights, leading to the conclusion that healthcare facility operators, despite facing financial and staffing constraints, should prioritise the following support measures, particularly during crises: comprehensive training, including resilience training; flexible work arrangements; and recognition for nursing staff. The study highlights the significance of personal and social resources in enhancing job satisfaction among nursing staff. Ultimately, healthcare organisations should aim to create a supportive work environment that cultivates a sense of community and belonging among their nursing workforce.

Additionally, healthcare facilities should directly consult nursing staff to identify their specific support needs. While hierarchical regression did not yield significant overall correlations to job satisfaction, descriptive analysis revealed a strong desire among nurses for increased personnel, financial, psychological and pastoral/palliative support.

## Figures and Tables

**Table 1:** Characteristics of the study population (n = 373).

		n	%
Age [mean, SD, range]		42.7, 12.8, 18–76	
Sex			
	Female	302	82.5
	Male	64	17.5
Professional qualification			
	Qualification as a nurse	188	52.6
	*(Geriatric nurse)*	*(142)*	*(40.0)*
	*(Nurse)*	*(35)*	*(9.9)*
	*(University diploma)*	*(11)*	*(3.1)*
	Nursing/geriatric nursing assistant	62	17.5
	In training	26	7.3
	Other qualification	41	11.5
	No qualification	38	10.7
Leadership position			
	No	276	76.7
	Yes	84	23.3

Missing: age n = 6, sex n = 7, Professional qualification n = 18, leadership position n = 13.

**Table 2:** Means and standard deviations of COPSOQ scales.

Variable	Number of Items	Cronbach’s Alpha	n	M	(SD)	Range
Job satisfaction	7	0.860	373	57.3	19.3	0–100
Social support	4	0.862	372	67.6	23.4	0–100
Sense of community at work	2	0.860	371	75.1	20.0	0–100
Commitment to the workplace	2	0.752	373	66.9	25.4	0–100
Recognition by management	1	-	367	50.4	29.3	0–100

**Table 3:** Association between organisational support measures and the job satisfaction scale.

	Yes			No					
	n	M	SD	n	M	SD	df	t	*p*
Regular updates about COVID-19 measures by superiors	316	59.0	18.2	40	44.04	22.4	354	−4.663	<0.001
Training programmes	252	60.2	17.9	81	47.8	20.4	331	−5.242	<0.001
Psychological support	100	61.4	18.7	225	54.8	18.8	323	−2.933	0.004
Pastoral and palliative support	144	61.5	18.9	187	53.6	19.0	329	−3.751	<0.001
Internal staff support	152	62.6	16.2	167	51.7	20.2	312.2	−5.336	<0.001
Changes in work organisation	139	64.8	15.3	168	51.5	19.8	303.8	−6.649	<0.001
In-house financial support	77	64.5	19.5	266	55.0	18.9	341	−3.865	<0.001
Relaxation of hygiene measures for vaccination protection	100	61.0	18.0	229	54.8	19.5	327	−2.693	0.007
Possibilities for communication between residents and relatives	240	60.0	18.0	102	50.0	20.0	340	−4.536	<0.001
Technical measures (e.g., use of air filters)	91	63.4	19.2	243	55.0	18.9	332	−3.589	<0.001

**Table 4:** Correlations of continuous variables.

	1	2	3	4	5	6
Dependent variable						
1. Job satisfaction	1	0.458 **	0.582 **	0.519 **	0.548 **	0.320 **
Independent variables						
2. Sense of community at work	0.458 **	1	0.559 **	0.353 **	0.298 **	0.115 *
3. Social support	0.582 **	0.559 **	1	0.454 **	0.495 **	0.220 **
4. Commitment to the workplace	0.519 **	0.353 **	0.454 **	1	0.489 **	0.154 **
5. Recognition by management	0.548 **	0.298 **	0.495 **	0.489 **	1	0.166 **
6. Resilience	0.320 **	0.115 *	0.220 **	0.154 **	0.166 **	1

Note. * *p* ≤ 0.05, ** *p* ≤ 0.01.

**Table 5:** Results of the regression analysis.

Model		b	SE	β	*p*	Confidence Interval		Corr. R^2^	∆R^2^
						Lower 95%	Upper 95%		
1	(Constant)	59.780	6.262		0.000	47.435	72.125	0.008	
	Age	−0.134	0.101	−0.095	0.186	−0.334	0.065		
	Sex	0.349	2.903	0.008	0.904	−5.373	6.072		
	Nursing/geriatric nursing assistant	−0.154	3.521	−0.003	0.965	−7.095	6.787		
	In training	−1.508	4.672	−0.023	0.747	−10.718	7.702		
	Other qualification	7.320	4.051	0.130	0.072	−0.666	15.306		
	No qualification	1.090	4.120	0.019	0.792	−7.033	9.213		
	Leadership position	5.957	2.816	0.156	0.036	0.406	11.509		
2	(Constant)	28.237	8.022		0.001	12.421	44.053	0.139 *	0.131
	Age	−0.142	0.094	−0.100	0.133	−0.328	0.044		
	Sex	0.373	2.704	0.009	0.891	−4.957	5.702		
	Nursing/geriatric nursing assistant	−0.051	3.279	−0.001	0.988	−6.516	6.413		
	In training	−0.921	4.352	−0.014	0.833	−9.501	7.659		
	Other qualification	8.124	3.775	0.145	0.033	0.681	15.567		
	No qualification	2.051	3.841	0.037	0.594	−5.521	9.624		
	Leadership position	2.220	2.703	0.058	0.412	−3.108	7.548		
	Resilience	10.598	1.851	0.377	0.000	6.949	14.247		
3	(Constant)	3.627	6.256		0.563	−8.709	15.963	0.559 *	0.420
	Age	−0.052	0.069	−0.037	0.451	−0.189	0.084		
	Sex	−2.278	1.967	−0.054	0.248	−6.156	1.600		
	Nursing/geriatric nursing assistant	0.370	2.361	0.008	0.875	−4.285	5.025		
	In training	1.263	3.129	0.019	0.687	−4.907	7.433		
	Other qualification	4.594	2.756	0.082	0.097	−0.839	10.028		
	No qualification	3.141	2.799	0.056	0.263	−2.377	8.660		
	Leadership position	2.102	1.952	0.055	0.283	−1.746	5.950		
	Resilience	5.270	1.386	0.188	0.000	2.538	8.002		
	Social support	0.140	0.046	0.178	0.003	0.049	0.231		
	Sense of community at work	0.206	0.048	0.226	0.000	0.111	0.301		
	Commitment to the workplace	0.102	0.039	0.145	0.009	0.025	0.178		
	Recognition by management	0.228	0.034	0.367	0.000	0.161	0.295		
4	(Constant)	3.619	6.249		0.563	−8.706	15.944	0.603 *	0.044
	Age	−0.041	0.068	−0.029	0.545	−0.176	0.093		
	Sex	0.484	1.963	0.012	0.805	−3.387	4.355		
	Nursing/geriatric nursing assistant	−1.267	2.299	−0.027	0.582	−5.801	3.268		
	In training	0.737	3.054	0.011	0.810	−5.287	6.761		
	Other qualification	2.608	2.683	0.046	0.332	−2.684	7.901		
	No qualification	1.187	2.758	0.021	0.667	−4.252	6.626		
	Leadership position	2.076	1.893	0.054	0.274	−1.658	5.810		
	Resilience	4.163	1.358	0.148	0.002	1.484	6.842		
	Social support	0.162	0.046	0.206	0.001	0.071	0.252		
	Sense of community at work	0.213	0.047	0.234	0.000	0.120	0.306		
	Commitment to the workplace	0.090	0.038	0.128	0.019	0.015	0.164		
	Recognition by management	0.204	0.034	0.329	0.000	0.137	0.272		
	Regular updates about COVID-19 measures	−9.414	3.056	−0.158	0.002	−15.442	−3.386		
	Training programmes	5.944	2.078	0.144	0.005	1.846	10.043		
	Psychological support	−0.759	2.064	−0.020	0.713	−4.829	3.311		
	Pastoral and palliative support	0.279	1.793	0.008	0.876	−3.258	3.816		
	Internal staff support	1.985	1.825	0.057	0.278	−1.614	5.584		
	In-house financial support	0.578	2.322	0.013	0.804	−4.002	5.158		
	Changes in work organisation	4.777	1.784	0.137	0.008	1.258	8.296		
	Relaxation of hygiene measures for vaccination protection	0.257	1.916	0.007	0.893	−3.523	4.037		
	Possibilities for communication between residents and relatives	−2.314	1.847	−0.062	0.212	−5.958	1.330		
	Technical measures	4.556	2.018	0.114	0.025	0.576	8.537		

* *p* < 0.001.

## Data Availability

The datasets presented in this article are not readily available because they are part of further analyses. The raw data supporting the conclusions of this article will be made available by the authors on request.

## Supplementary Materials

The following supporting information can be downloaded at: https://www.mdpi.com/article/10.3390/geriatrics9060159/s1, Figure S1: Quantity of organisational measures available in each support category.
