# Supplementary figures and images for: Supporting Nursing Staff During Crises: Impact of Organisational Support Measures and Resources in Job Satisfaction in German Nursing Homes
Source: Geriatrics (Basel). 2024 Dec 11;9(6):159. doi: 10.3390/geriatrics9060159 (PMC11728096; doi:10.3390/geriatrics9060159)

Figure S1: Quantity of organisational measures available in each support category.

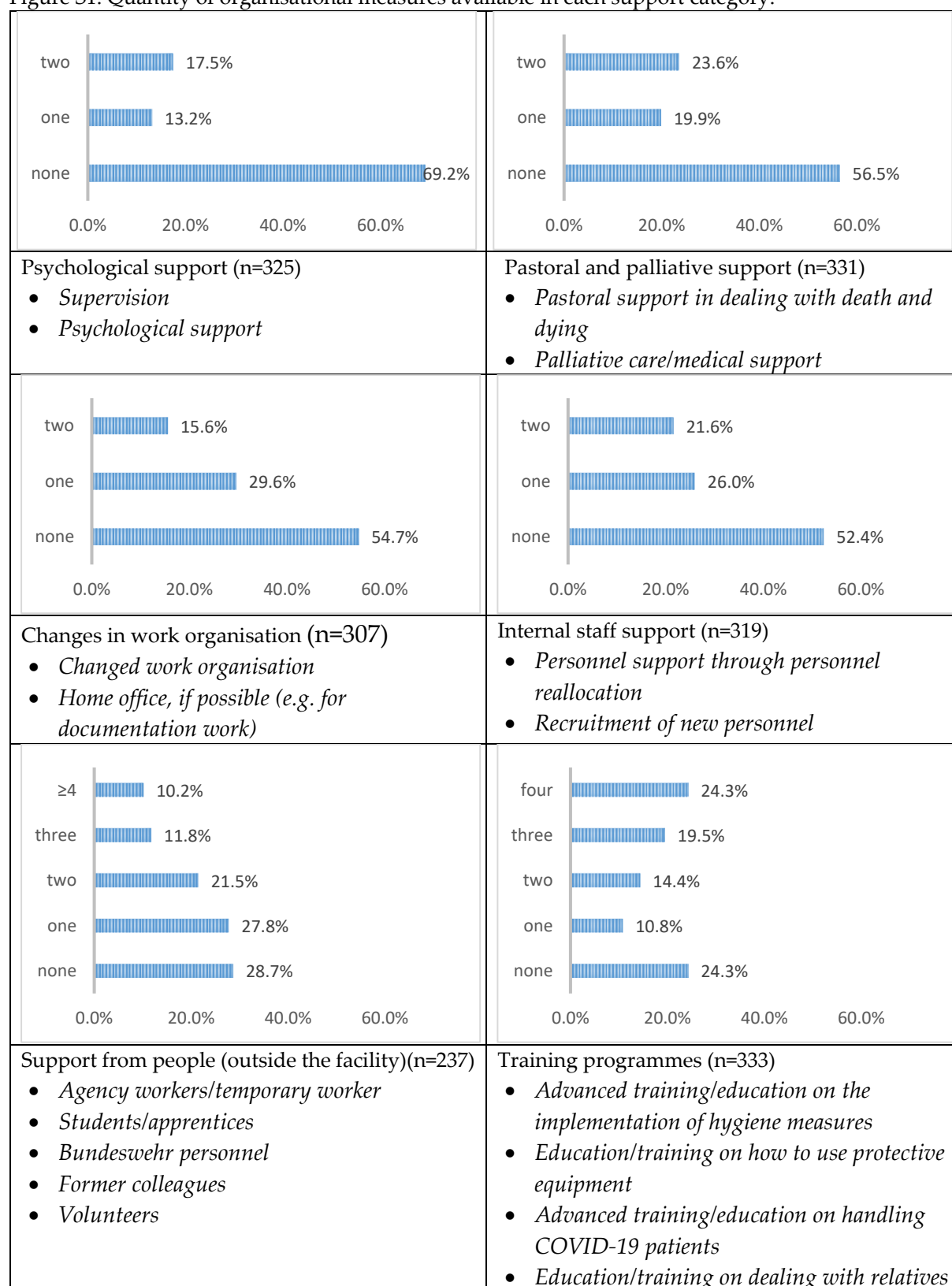

Supplement: Supplementary file 1 [file geriatrics-09-00159-s001.zip › geriatrics-3356847-supplementary.pdf]
